# Gut microbiota and metabolic marker alteration following dietary isoflavone‐photoperiod interaction

**DOI:** 10.1002/edm2.190

**Published:** 2020-10-17

**Authors:** Mario G. Oyola, Ryan C. Johnson, Bradly M. Bauman, Kenneth G. Frey, Ashley L. Russell, Madelaine Cho‐Clark, Katelyn N. Buban, Kimberly A. Bishop‐Lilly, D. Scott Merrell, Robert J. Handa, T. John Wu

**Affiliations:** ^1^ Department of Obstetrics and Gynecology Uniformed Services University of the Health Sciences Bethesda MD USA; ^2^ Center for Neuroscience and Regenerative Medicine Uniformed Services University of the Health Sciences Bethesda MD USA; ^3^ Henry M. Jackson Foundation for the Advancement of Military Medicine Bethesda MD USA; ^4^ Genomics and Bioinformatics Department Biological Defense Research Directorate Naval Medical Research Center – Frederick Fort Detrick MD USA; ^5^ Program in Emerging Infectious Diseases Uniformed Services University of the Health Sciences Bethesda MD USA; ^6^ Department of Microbiology and Immunology Uniformed Services University of the Health Sciences Bethesda MD USA; ^7^ Department of Biomedical Sciences Colorado State University Fort Collins CO USA

**Keywords:** circadian rhythms, gut brain axis, isoflavones, metabolism, microbiome, obesity

## Abstract

**Introduction:**

The interaction between isoflavones and the gut microbiota has been highlighted as a potential regulator of obesity and diabetes. In this study, we examined the interaction between isoflavones and a shortened activity photoperiod on the gut microbiome.

**Methods:**

Male mice were exposed to a diet containing no isoflavones (NIF) or a regular diet (RD) containing the usual isoflavones level found in a standard vivarium chow. These groups were further divided into regular (12L:12D) or short active (16L:8D) photoperiod, which mimics seasonal changes observed at high latitudes. White adipose tissue and genes involved in lipid metabolism and adipogenesis processes were analysed. Bacterial genomic DNA was isolated from fecal boli, and 16S ribosomal RNA sequencing was performed.

**Results:**

NIF diet increased body weight and adipocyte size when compared to mice on RD. The lack of isoflavones and photoperiod alteration also caused dysregulation of lipoprotein lipase (*Lpl*), glucose transporter type 4 (*Glut‐4*) and peroxisome proliferator‐activated receptor gamma (*Pparg*) genes. Using 16S ribosomal RNA sequencing, we found that mice fed the NIF diet had a greater proportion of Firmicutes than Bacteroidetes when compared to animals on the RD. These alterations were accompanied by changes in the endocrine profile, with lower thyroid‐stimulating hormone levels in the NIF group compared to the RD. Interestingly, the NIF group displayed increased locomotion as compared to the RD group.

**Conclusion:**

Together, these data show an interaction between the gut bacterial communities, photoperiod length and isoflavone compounds, which may be essential for understanding and improving metabolic health.

## Graphical abstract

Isoflavones are known to shift the metabolic state. In this study, we found that this effect is accentuated during short active photoperiods. Removal of isoflavones shifted the microbiome composition to one that resembles that observed in obese models, accompanied by increased adipocyte size and the expression of markers of metabolic dysfunction

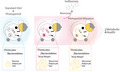

## 1 INTRODUCTION

Obesity is an epidemic affecting one in three Americans.^1^ While the risk of developing obesity is higher in females, males are more prone to developing obesity‐related diseases, including nonalcoholic fatty liver disease, insulin resistance, type 2 diabetes, and cardiovascular pathology.^2^ Growing evidence indicates that soy isoflavones derived have beneficial roles in reducing obesity and diabetes markers.^3^, ^4^ However, the precise mechanisms involved remain unknown.

Isoflavones are metabolized into active compounds by the host gut microbiota.^5^ While estrogens and the gut bacterial communities are known to play independent roles in obesity, new research suggests that estrogenic metabolites and the microbiota may work together to regulate metabolism.^5^, ^6^ For example, certain microbiota convert isoflavones to biologically active compounds, and in return, isoflavones may permit specific microbiota to thrive, resulting in their proliferation and marked growth. Thus, microbes are essential for the regulation of food's nutritional and energetic benefits, and their diversity is highly correlated with energy balance.^7^

The gut microbiota composition and diversity differ between lean and obese profiles. For example, obese mice have a higher relative abundance of bacteria within the Firmicutes phylum compared to the Bacteroidetes phylum.^8^ Furthermore, if the fecal matter containing the microbiota of obese mice is cross‐transplanted into germ‐free lean mice, the lean animals become more obese than the ones transplanted with fecal matter from lean mice.^8^ Together, these reports strongly suggest that certain gut microbial profiles are associated with obesity.^9^, ^10^

While many other factors influence the gut microbiome, circannual rhythm is one that not only influences the microbiota composition,^11^, ^12^, ^13^ but also impacts metabolism.^14^ Fluctuations in environmental cues, mainly driven by the length of the active vs inactive periods, have been linked to changes in the gut microbiota.^11^, ^12^, ^13^ In winter, when active periods are shorter, the number of human subjects that meet the criteria for metabolic syndrome is significantly higher.^15^ In the same study, the authors concluded that short active periods exacerbate metabolic pathology.

Photoperiod interaction with dietary isoflavones may play a critical role in regulating most physiological functions by closely influencing the gut microbiota.^16^ While several studies have established the respective effects of diet and photoperiod on the gut microbiome, a gap in knowledge remains as to how these two environmental cues may ultimately interact to affect the gut microbiome. In this study, we manipulated isoflavone consumption and photoperiod duration to examine how these external cues interact to dictate the temporal stability of the gut microbiome and its relationship with obesity and metabolism.

## 2 METHODS

### 2.1 Animals

Male C57BL/6J mice, aged 8 weeks old upon arrival, were obtained from The Jackson Laboratory (Stock No. 000664). All animals were housed in groups of four animals per cage and maintained on a 12‐hour light and 12‐hour dark light cycle (lights off 1300 hours) under controlled temperature conditions (22 ± 1°C) with ad libitum access to standard rodent chow and water throughout the experiment. Behavioural testing was conducted between 0600 and 1000 hours, and animals were acclimated to the behavioural room at 0600 hours for an hour before testing at 0700 hours. Cage changes, which coincides with fecal boli collection, were performed between 0700 and 0900 hours. Handling and care of animals were conducted per the National Institutes of Health Guide for Care and Use of Laboratory Animals and approved by the Institutional Animal Care and Use Committee at the Uniformed Services University of the Health Sciences, Bethesda, Maryland.

### 2.2 Diet

Following 2 weeks of acclimation to the facility and standard rodent chow (2018 Teklad Global 18% Protein Rodent Diet; Envigo), animals were randomly assigned to each experimental group (RD or NIF, 12L:12D or 16L:8D photoperiods). Mice in the first group assignment (diet) were randomly designated to the standard chow (maintained on the RD) or switched to an isocaloric isoflavone‐free (NIF) chow (AIN‐93G Purified Rodent Diet with corn oil replacing soybean oil; Dyets). The AIN‐93 diet did not differ in macronutrient, mineral and vitamin composition from the Teklad Global 18% Protein Rodent Diet.^17^ Both diets were previously characterized by our laboratory for isoflavone content using high performance liquid chromatography based analysis according.^16^, ^18^, ^19^ The standard chow contains a total of 199.4 μg/g of isoflavone equivalents, whereas the NIF diet contains 0 μg/g of isoflavone equivalents.^18^ All experimental groups were acclimated to these diets for 3 weeks prior to photoperiod modification.

### 2.3 Photoperiod

Following the diet acclimation, mice were randomly assigned into a 12‐hour light on, 12‐hour light off (12L:12D) photoperiod (lights on at 1300 hours), or to a shortened activity period, 16‐hour lights on, 8‐hour lights off (16L:8D); lights on at 2100 hours). All animals assigned to the 16L:8D group were switched to an identical neighbouring room where lights were adjusted accordingly (see Figure S1 for timeline).

### 2.4 Tissue collection

Trunk blood was collected following deep anesthetization with CO_2_ inhalation and rapid decapitation. Blood was allowed to clot on ice and spun at 2000 *g* for 10 minutes prior to serum collection. The serum was then stored at −80°C until assayed.

### 2.5 White adipose tissue histology

Epididymal white adipose tissue (eWAT) and livers were collected. Samples were stored at −80°C until processed. While frozen, eWAT was divided in half. One half of the frozen tissue was fixed in 10% buffered formalin at 4°C for 48 hours.^20^ The other half was immediately immersed in Ribozol for RNA extraction (see below RNA section). Once fixed, a total of four randomly selected samples per group were paraffin‐embedded and sliced at 5 µm using a microtome (Microm HM 355S; Thermo Scientific). Three consecutive sections per paraffin block were cut and mounted per slide. A total of three sections per animal were processed and analysed (n = 4 animals/group). Standard haematoxylin and eosin staining was performed. Tissue was scanned using the Carl Zeiss Axioscan Z1 (10×/0.45, M27 Plan Apochromat objective lens) and the Carl Zeiss Zen Blue (2012) software.

### 2.6 Adipocyte analysis

Adipocyte areas were measured using ImageJ software and the Adiposoft plugin^21^, ^22^ by an investigator blinded to treatment group. Three random fields per sample were analysed. The average area per field of all measured adipocytes was used to calculate the adipocyte area. An average of 3000 cells per field was analysed (n = 4/group). The frequency of adipocytes was calculated by combining all areas of measured cells per respective group and then distributing across four bins from 1000 to 4000 μm.

### 2.7 RNA extraction and isolation

Total RNA from the white adipose and liver tissue was extracted using Ribozol RNA Extraction Reagent (Cat. No. N580; AMRESCO) by homogenization in 1 mL of Ribozol followed by RNA purification and isolation according to the manufacturer's protocol. DNase digestion was performed after RNA purification using the Direct‐zol RNA Miniprep Kit (Cat. No. R2052; Zymo Research) according to the manufacturer's protocol, including on‐column DNase digestion. The concentration and quality of the RNA were determined using a NanoDrop Lite spectrometer (Thermo Fisher). Total RNA was reverse‐transcribed to single‐stranded cDNA using the Maxima First Strand cDNA Synthesis Kit (Cat. No. K1642; Thermo Fisher). (n = 6/group).

### 2.8 Quantitative real‐time PCR

The mRNA expression of peroxisome proliferator‐activated receptor gamma (*Pparg*), nuclear factor erythroid 2 p45‐related factor 2 (*Nrf2*), and the control gene TATA‐box binding protein (*Tbp*) was assessed in each liver (n = 6/group) sample. For the white adipose tissue (n = 5‐6/group), lipoprotein lipase (Lpl), glucose transporter type‐4 (Glut‐4), Pparg, CD36 and TBT were assayed. All mRNA expression was assayed via quantitative real‐time PCR using the iQ SYBR Green Supermix (cat. no. 1708884; Bio‐Rad). Primer sequences for white adipose tissue and liver can be found in the Tables 1 and 2, respectively. Each sample was assayed in triplicate using 400 nmol/L of the respective primer pair on the CFX Connect Real‐time System (Bio‐Rad). The following cycling parameters were used: initial denaturation and enzyme activation at 95°C for 3 minutes followed by 40 cycles of denaturation (95°C, 15 seconds), annealing (60°C, 30 seconds), extension (72°C, 30 seconds), and reading. Melt curve analysis was conducted after each real‐time reaction to demonstrate the presence of a single amplicon. Relative expression of each gene was determined using the delta delta *C*_t_(ΔΔ*C*_t_) method,^23^, ^24^ normalizing each sample to *Tbp*.

**TABLE 1:** White adipose tissue qRT‐PCR genes

Gene	Accession number	Primer sequence	Amplicon size (bp)
*Lpl*	NM_008509.2	(F) 5′‐ATGGATGGACGGTAACGGGAATGT‐3′	128
		(R) 5′‐TGGATAATGTTGCTGGGCCCGATA‐3′	
*Glut‐4*	NM_009204.2	(F) 5′‐GTAACTTCATTGTCGGCATGG‐3′	155
		(R) 5′‐AGCTGAGATCTGGTCAAACG‐3′	
*Pparg*	NM_011146.3	(F) 5′‐CCACCAACTTCGGAATCAGCT‐3′	434
		(R) 5′‐TTTGTGGATCCGGCAGTTAAGA‐3′	
*CD36*	NM_007643.4	(F) 5′‐TCCTCTGACATTTGCAGGTCTATC‐3′	99
		(R) 5′‐AAAGGCATTGGCTGGAAGAA‐3′	
*Tbp*	NM_013684.3	(F) 5′‐CCTATCACTCCTGCCACACC‐3′	161
		(R) 5′‐ATGACTGCAGCAAATCGCTTG‐3′	

**TABLE 2:** Liver qRT‐PCR genes

Gene	Accession number	Primer sequence	Amplicon size (bp)
*Tbp*	NM_013684.3	(F) 5′‐CCTATCACTCCTGCCACACC‐3′	161
		(R) 5′‐ATGACTGCAGCAAATCGCTTG‐3′	
*Nrf2*	NM_010902.4	(F) 5′‐AGTGGATCCGCCAGCTACT‐3′	122
		(R) 5′‐GCAAGCGACTCATGGTCATCTAC‐3′	
*Pparg*	NM_011146.3	(F) 5′‐TCAGGCTTCCACTATGGAGTTC‐3′	195
		(R) 5′‐CCCAAACCTGATGGCATTGTGA‐3′	

### 2.9 Open field arena (OFA)

We assessed locomotion at three time points: prior to any experimental manipulation (baseline/OFA1), following diet change (OFA2), and after photoperiod alteration (OFA3). The open field apparatus was 40 cm × 40 cm (Stoelting Co.) with gray floor and opaque walls. Mice (n = 12/group) were brought to the behavioural core and allowed to acclimate for at least 60 minutes before the behavioural assessment. Each animal was individually placed in the centre of the apparatus and allowed to freely move for 30 minutes. Behaviours were video recorded and tracked using the ANY‐maze software (Stoelting Co.). All OFAs were illuminated with approximately 175 lux.^25^ Fecal boli were counted at the end of the 30 minutes. 70% ethanol was used to clean the apparatus between animals after fecal boli deposits were visually counted and numbers recorded. The total distance (locomotor activity) and immobile time were recorded.

### 2.10 Elevated plus maze

Two days after the last open field assessment, animals (n = 12/group) were tested in the elevated plus maze (EPM). The EPM was constructed of Plexiglas with two open arms (35 × 5 cm) and two enclosed black arms (35 × 5 × 16 cm) at an elevation of 50 cm above the floor. Mice were placed in the centre of the junction of the arms of the maze facing an open arm, and the behaviour was analysed for 5 minutes. Illumination was approximately 1600 Lux in the open arm and 200 Lux in the closed arm.^25^ The number of entries into the open and closed arms and the time spent exploring the open and closed arms were recorded and analysed using the ANY‐maze software (Stoelting Co.). The time spent in the centre platform (5 cm × 5 cm) not exploring any of the arms was not included in the calculations. The maze was cleaned with 70% ethanol solution after each session and allowed to dry between the sessions. No significant differences were detected in any of the EPM measurements (data not shown).

### 2.11 Fecal boli

Fecal content was collected (n = 12/group) at four different time points, as depicted in Figure S1. The first collection (baseline) took place 2 weeks after the animals' arrival to the university facility and acclimation to the regular diet. Changes driven by the isoflavone‐free diet were determined by the second collection, which took place 2 weeks following the diet switch. Both of these collections were performed during the scheduled weekly cage change. Photoperiod‐dependent changes were assessed on the third collection, and it was performed at the end of the open field test. The combined effect of diet and photoperiod alteration was tested on the fourth collection, which took place during the sacrifice. All collections were stored at −80°C until processed. Bacterial genomic DNA (gDNA) was isolated from the pellets using the DNeasy PowerSoil HTP 96 Kit (Cat. No. 12955‐4; Qiagen) per the manufacturer's protocol.

### 2.12 Microbiome analysis

The V4 region of the 16S rRNA gene was PCR amplified from the extracted gDNA using the 515F and 806R primers (https://doi.org/10.1128/mSystems.00009‐15) in accordance with the 16S Illumina amplicon protocol from the Earth Microbiome Project (http://press.igsb.anl.gov/earthmicrobiome/protocols‐and‐standards/16s/). In total, we sequenced 192 samples (n = 12/group) on the Illumina MiSeq platform using paired‐end 300 bp sequencing (v3 600 cycle kit, catalog number MS‐102‐3003) which yielded a total of 15 966 081 paired reads. On average, each sample contained approximately 83 157 reads (range = 21 146‐132 073). The reads were quality filtered, merged, and chimera checked using DADA2 (v1.10.1) (https://doi.org/10.1038/nmeth.3869). In brief, forward and reverse reads were trimmed to 200 and 150 base pairs, respectively, and further trimmed to the first instance of a quality score less than 2. All reads that contained ambiguous base calls (N's) or had greater than 2 expected errors were removed from the study. This filtering removed approximately 29.2% (4 667 053) of the reads from the analysis which resulted in an average of 58 849 reads per sample (range = 17 075‐91 422).

DADA2 was also used to assign taxonomy to each amplicon sequence variant (ASV) using the Silva non‐redundant database (v132). Reads associated with ASVs that were present in less than 5% of the samples were removed from the analysis. Additionally, reads with either no phylum level classification or were associated with the Cyanobacteria phylum were removed due to low sample prevalence. The final reads per ASV data for each fecal sample is provided in Table S1. Relative abundance, alpha diversity metrics, distance matrix calculations, and ordination analyses were performed using the Phyloseq R package (v1.30.0).^26^ We used the analysis of variance of distance matrices test (Adonis) to detect significant differences in ordination clusters using the vegan R package (v2.5‐6). Furthermore, ASV relative abundance data were used as input for predictive modelling using the random forest algorithm (ranger R package v0.12.1) in order to extract ASVs that were predictive of diet and light groups. Models were trained using 75% of the mouse samples either post‐diet change (time points 2‐4) or post‐light change (time points 3 and 4). Models were tested on the remaining 25% of the data. All microbiome data and statistical analyses were conducted within the R programming language (v3.6.1).^27^

### 2.13 Serum hormones

Previously frozen serum was thawed on ice and assayed for serum hormone levels. Serum prolactin, GH, and TSH levels (n = 5‐6/group) were determined using the MILLIPLEX Mouse Pituitary Magnetic Bead Panel (Cat. No. MPTMAG‐49K; MilliporeSigma) per the manufacturer's protocol. The interassay coefficient of variance (CV) was 6.14%.

### 2.14 Statistics (non‐microbiome)

Non‐microbiome‐related statistical analyses were performed on GraphPad PRISM 8. Data are presented as mean ± standard error of the mean (SEM). Adipocyte area, hormones, behaviours, qRT‐PCR, and fecal boli quantification were analysed using 2‐way analysis of variance (ANOVA) followed by Sidak's multiple comparison test when significant interaction (*P* < .05) between diet and photoperiod were detected. Pre‐photoperiod body weight was analysed using a 2‐way ANOVA with repeated measures and post‐photoperiod using 3‐way ANOVA with repeated measures, both followed by appropriate post hoc tests (further described in results). Threshold for statistical significance was set at *P* < .05.

## 3 RESULTS

### 3.1 Weight gain following isoflavone‐free diet and photoperiod interaction

**FIGURE 1:**
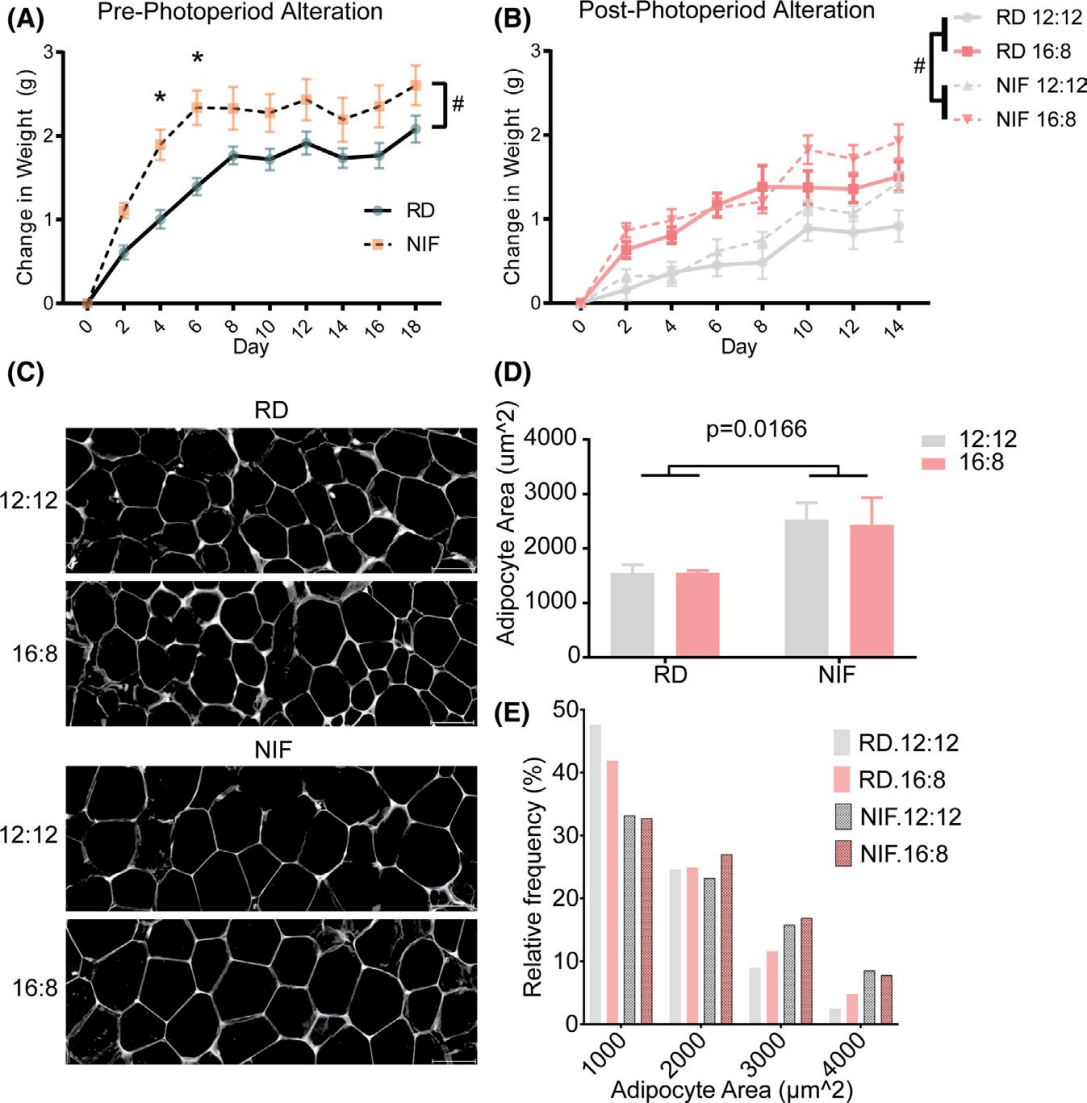
Body weight and adipocyte size change following isoflavone‐free diet and short activity photoperiod. A, Isoflavone‐free diet causes increase in body weight. B, Body weight following photoperiod alteration. C, Representative images of H&E staining of eWAT sections; scale bar = 50 μm. D, Reduction in mean adipocyte cell surface area in mice in NIF diet. E, Distribution frequency of cell size (bins of 1000 μm^2^). Adipocyte size data are expressed as mean ± standard error of the mean. Adjusted *P* values are shown. Regular (RD) and No Isoflavone (NIF) diet groups

We tracked the body weight of every animal from their arrival to the end of the protocol. For analysis purposes, we split the weight measurements into pre‐ and post‐photoperiod alterations (Figure 1A,B). A 2‐way ANOVA with repeated measures of the pre‐photoperiod data showed that the lack of isoflavones led to increased weight gain when compared to animals on the regular diet (*P* < .05). Sidak's multiple comparisons test revealed a significant change at days 4 (*P* < .005) and 6 (*P* < .005). The weight gain observed in animals on the IF diet was exacerbated by the longer inactive period (Figure 1B). A 3‐way ANOVA revealed a significant effect of diet (*P* < .0001) and photoperiod (*P* < .0001). The interaction between diet and photoperiod was also significant (*P* < .05). Tukey's multiple comparison analysis showed significant differences at various time points. Our previous work suggests that the increase in body weight is independent of the amount of food consumed, as the animals in the NIF diet have increased body weight despite consuming less food.^18^

### 3.2 Isoflavone content defines white adipose tissue metabolic state

Histological observations revealed a marked difference in the white adipose tissue (Figure 1C). A 2‐way ANOVA revealed a significant effect of diet (*F*_1,11_ = 7.964, *P* < .05). The adipocyte area of animals on the NIF diet was significantly larger than that of animals on the regular diet (Figure 1C‐E). The animals in the RD group had a higher frequency of adipocytes with an area less than or equal to 1000 μm^2^. However, the frequency of adipocytes in the 3000 μm^2^ bin centre was higher (frequency = RD, 12L:12D = 8.94, RD, 16L:8D = 11.60, NIF, 12L:12D = 15.80, NIF, 16L:8D = 16.92) in animals on the NIF diet when compared those on the RD (Figure 1E).

### 3.3 Isoflavone content and length of photoperiod alter white adipose tissue metabolism markers

**FIGURE 2:**
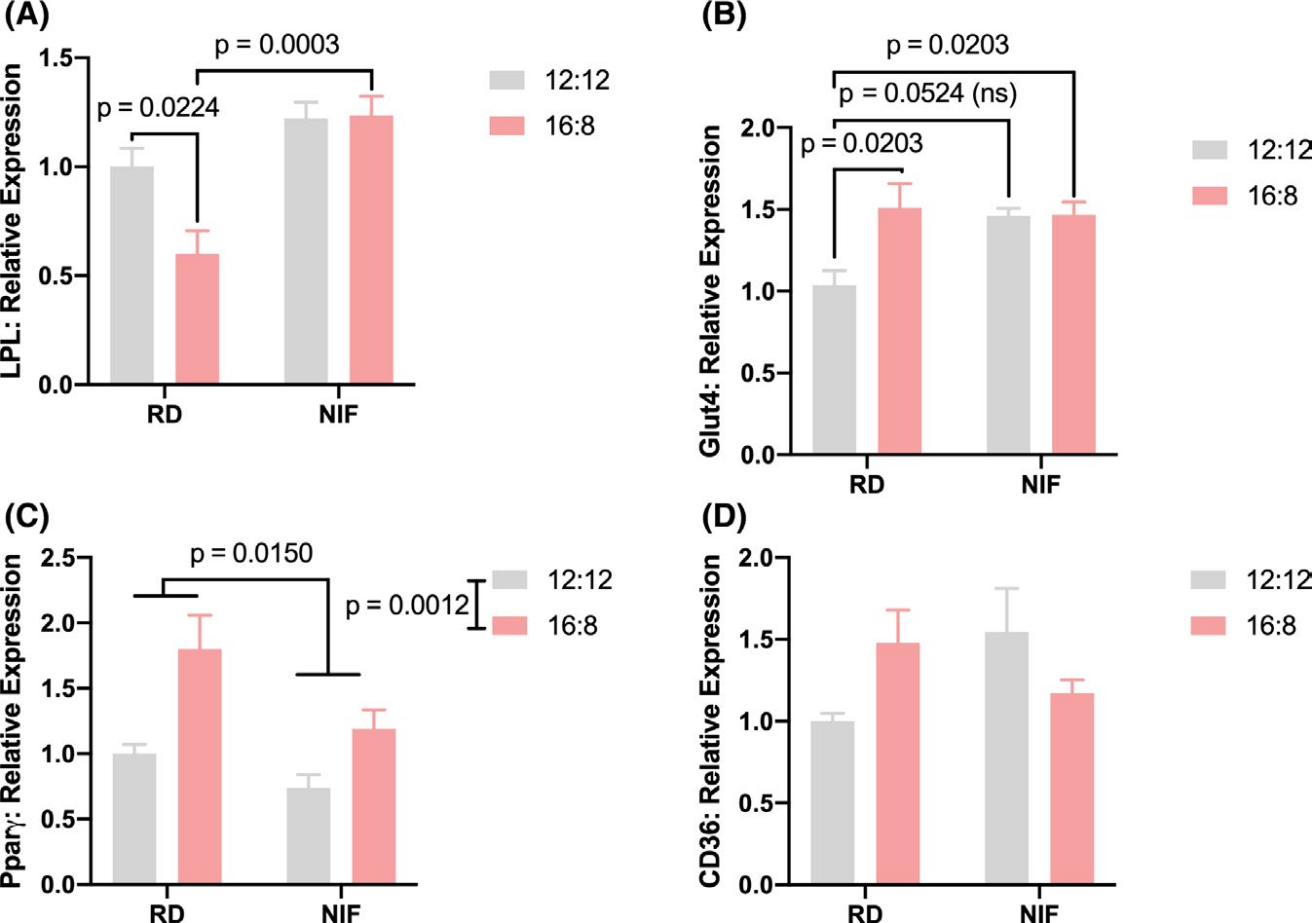
White adipose tissue metabolism markers. RNA transcript levels of (A) lipoprotein lipase (*LPL*), (B) glucose transporter type 4 (*Glut4*), (C) peroxisome proliferator‐activated receptor gamma (*Pparg*), and (D) CD36 were determined via qRT‐PCR. Data are expressed as mean ± standard error of the mean. Adjusted *P* values are shown (ns, not significant). Regular (RD) and No Isoflavone (NIF) diet groups

To investigate the potential cause of the adipose tissue remodelling and body weight increase, we assessed the effect of isoflavones and photoperiod length on genes involved in the regulation of adipocyte metabolic function, proliferation and differentiation (Figure 2A‐D). Consistently with the increase in body weight and adipocyte size in the NIF treated animals, *Lpl* mRNA transcript levels were significantly higher (*F*_1,20_ = 23.29, *P* < .0001) when compared to those in the RD groups as revealed by 2‐way ANOVA (Figure 2A). Furthermore, the groups in the 16L:8D photoperiod had lower (*F*_1,20_ = 4.70, *P* < .05) *Lpl* transcript levels when compared to the 12L:12D groups. There was also a significant (*F*_1,20_ = 5.43, *P* < .001) interaction between diet and photoperiod duration on *Lpl* levels. Post hoc analysis revealed that animals within the 16L:8D groups, *Lpl* transcripts were significantly lower (*P* < .001) in the NIF group when compared to the animals in the regular diet. Additionally, within the regular diet groups, animals in the 16L:8D group had significantly lower (*P* < .001) *Lpl* mRNA transcripts when compared to the 12L:12D photoperiod.

The *Glut‐4* mRNA transcript levels were elevated following diet and photoperiod modifications when compared to animals in the 12L:12D RD (Figure 2B). A 2‐way ANOVA revealed a significant effect of photoperiod (*F*_1,18_ = 5.474, *P* < .05), but no significant effect of diet (*F*_1,18_ = 3.449, *P* > .05). However, there was a diet × photoperiod interaction effect (*F*_1,18_ = 5.211, *P* < .05). Within the regular diet groups, *Glut‐4* transcript levels were higher (*P* < .05) in the 16L:8D group compared to the animals in the 12L:12D group. The NIF diet group in the 16L:8D photoperiod had higher *Glut‐4* mRNA transcript levels when compared to the animals in the regular diet in the 12L:12D photoperiod.

Similarly, animals in the 16L:8D groups had elevated (*F*_1,17_ = 15.19, *P* < .01) *Pparg* transcript levels when compared to the animals in the 12L:12:D groups, as revealed by a 2‐way ANOVA (Figure 2C). However, there was an effect of diet as reflected by a decrease (*F*_1,17_ = 7.33, *P* < .05) of these transcript levels in the NIF groups when compared to the regular diet. No photoperiod and diet interaction was detected *F*_1,17_ = 1.17, *P* > .05. Finally, no significant differences were detected on *CD36* mRNA transcripts (Figure 2D).

### 3.4 Isoflavone content and length of photoperiod alter gut microbiota composition

**FIGURE 3:**
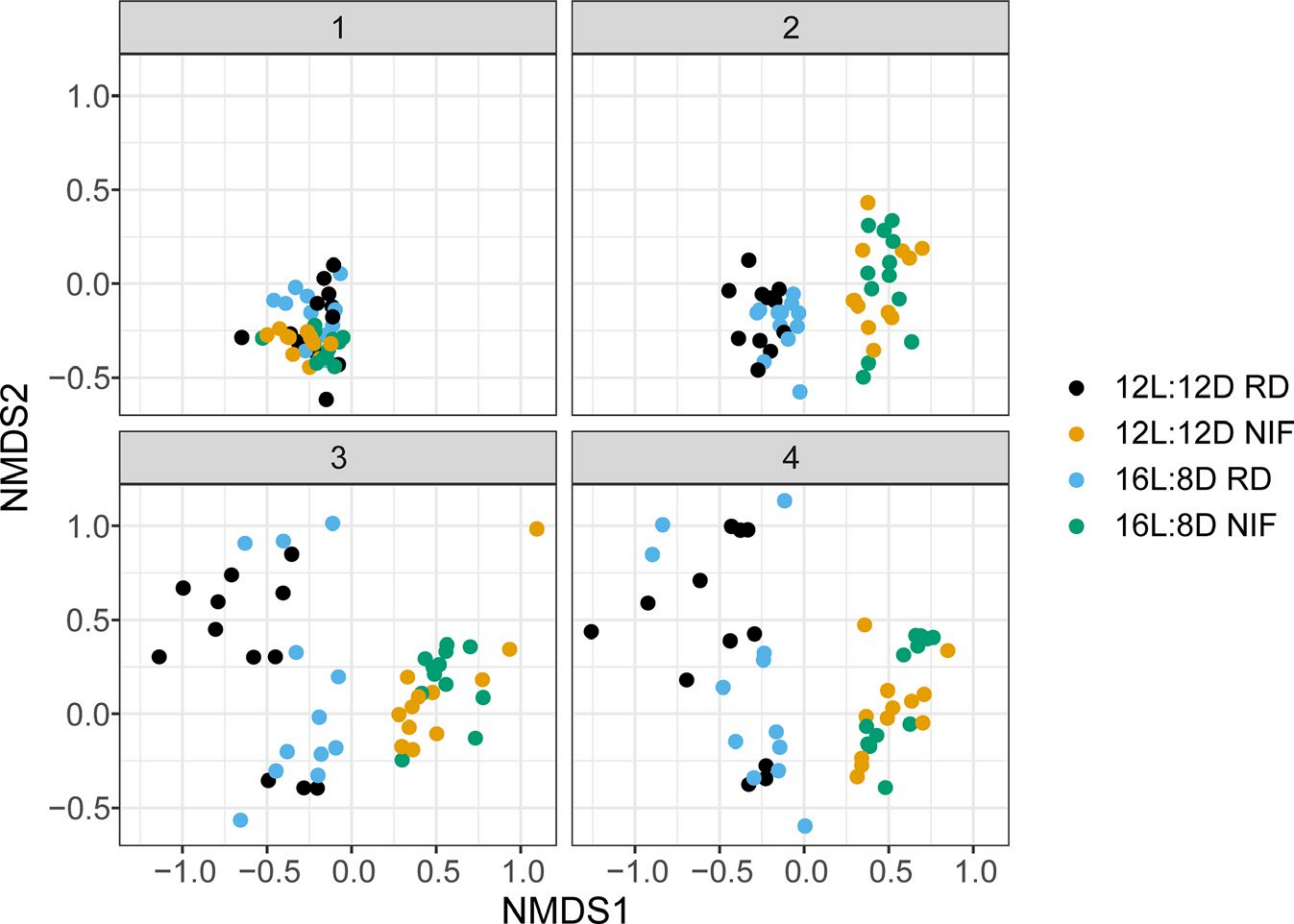
NMDS of fecal microbiome samples by time point and study group. Non‐metric multidimensional scaling (NMDS) was performed on a Bray‐Curtis dissimilarity matrix that utilized ASV relative abundance as input. Each point corresponds to an individual fecal microbiome sample. The closer any two points are to each other, the more similar they are compositionally. The plots are separated by sample time point (1, 2, 3 or 4) and colour coded based on diet and photoperiod groups. 12L:12D, 12 h light and 12 h dark; 16L:8D, 16 h light and 8 h dark; NIF, isoflavone‐free diet; RD, regular diet

To assess whether the NIF diet and photoperiod length induced changes in the fecal microbiota, we performed targeted 16S rRNA sequencing of gDNA samples obtained from animals in the diet and photoperiod protocols. While most microbiome samples were indistinguishable at time point 1, we observed distinct clustering of the two diet groups (NIF and RD) for the remainder of the study (time points 2, 3, and 4) (Adonis, *P* < .001) (Figure 3). However, the effect of photoperiod on microbial composition was less pronounced; only the NIF group at time point 2 showed a significant difference in clustering between the 12L:12D and 16L:8D groups (Adonis, *P* = .019).

**FIGURE 4:**
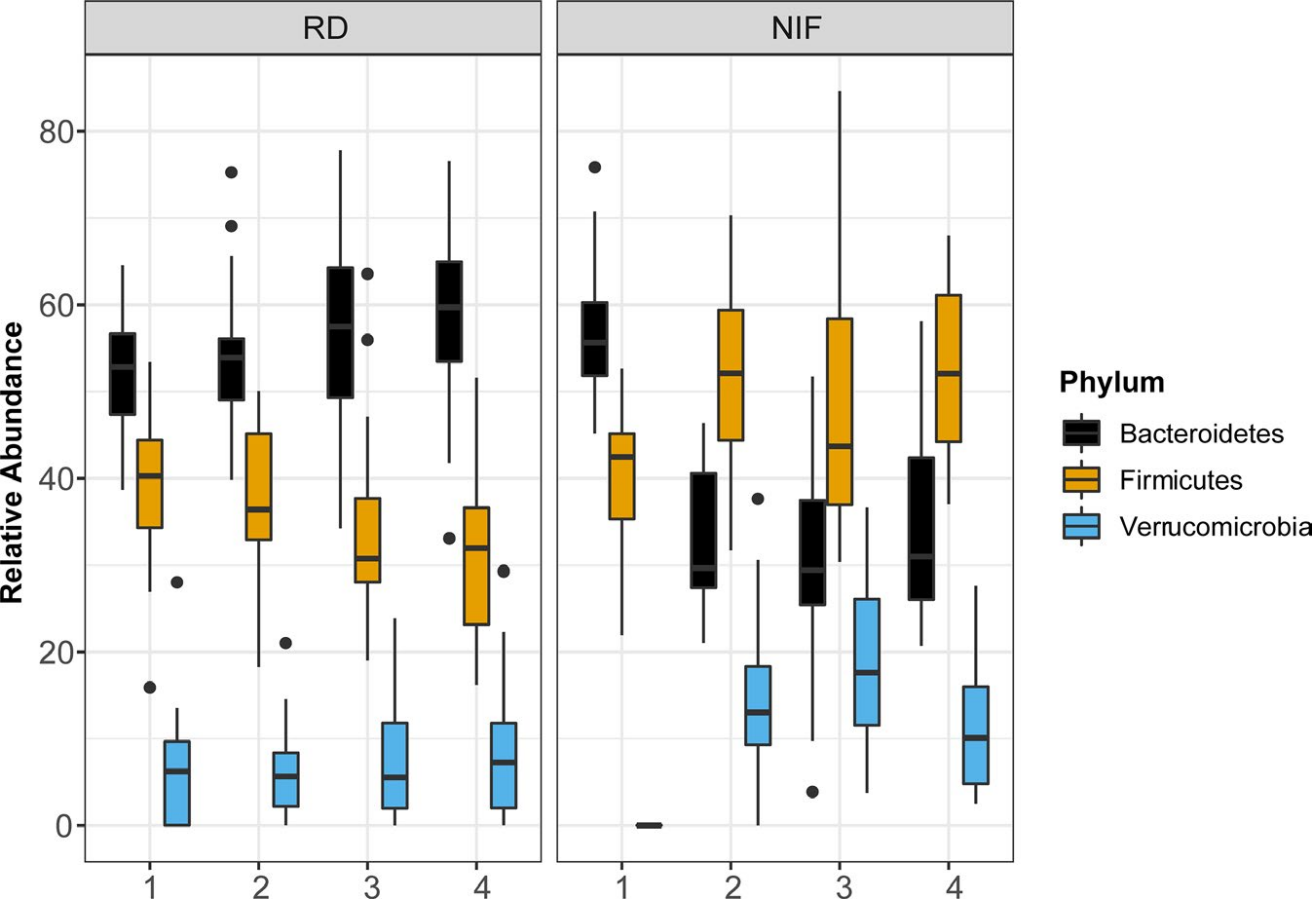
Relative abundance of the top three most abundant phyla over time. Data are split into regular (RD) and isoflavone‐free (NIF) diet groups. Each box plot summarizes the distribution of the relative abundance for the three phyla depicted with the centre line representing the median, the box edges are the 25% and 75% quantiles, and the whiskers extend to the minimum and maximum values. Values outside 1.5 times the interquartile range (outliers) are plotted as dots. The relative abundance for each phylum is displayed for each sample point (1, 2, 3 and 4)

At the bacterial Phylum level, we observed a switch in the levels of Bacteroidetes and Firmicutes in response to the diet implementation (Figure 4). The NIF diet induced an increase in the Firmicutes and a decrease in Bacteroidetes. Verrucomicrobia was also increased in the NIF diet but not in the RD. The top six most abundant phyla for each individual animal can be observed in Figure S2.

We next used machine learning to extract genera that were most important in differentiating between the RD and NIF groups. We found that our random forest model was able to predict diet group with 100% accuracy. The top 10 most predictive ASVs are shown in Figure S3. We found that much of the variation between the RD and NIF diet groups was attributed to ASVs within the Lachnospiraceae and Clostridiales family of bacteria. We also found that the single most predictive ASV belonged to the Muribaculaceae family which was highly abundant in the RD group (~5%‐30% relative abundance), and was nearly ablated from the gut in the NIF group (<5% relative abundance). At the genus level, we found that the NIF diet resulted in decreased abundance of *Lactobacillus*, *Rumminococcus*, genus group NK4A136 of the Lachnospiraceae family, and the UCG‐014 genus of the Ruminiococcaceae family. Conversely, we observed and increased abundance of *Dubosiella*, *Acetatifactor*, and *Bifodbacterium* (Figure S4). The relative abundance of the top 30 genera is available in Figure S5, for means and Figure S6 for individual animals.

Although we did not observe a major effect on microbial composition due to altered photoperiod within the RD or NIF diet groups (Adonis, *P* > .05, time points 3 and/or 4) (Figure 3), we were able to extract ASVs that were predictive of photoperiod group with 100% accuracy in the RD group and 91.7% accuracy for the NIF group. The top 10 predictive ASVs of photoperiod group within each diet group are shown in Figure S7. Overall, the majority of photoperiod‐predictive ASVs were low in relative abundance (<2% relative abundance) and often absent in the samples. We additionally did not observe overt differences between photoperiod groups among the top 30 most abundant genera in our study (Figures S5 and S6).

Finally, we assessed alpha diversity levels within each diet and photoperiod group. For all diversity metrics tested (Observed ASVs, Shannon, Inverse Simpson), we observed a drop in diversity once isoflavones were removed from the diet (NIF) compared to the RD group (time points 2‐4 RD diet vs time points 2‐4 NIF diet, two‐tailed *t* test *P* < .001, Figure S8). With respect to photoperiod, we detected a difference in the observed ASVs for the IF diet group between the 12L:12D and 16L:8D groups (time points 3 & 4 12L:12D vs time points 3 & 4 16L:8D, two‐tailed *t* test, *P* = .033). This suggests that within the IF diet group, increased light exposure results in a drop in ASVs detected (richness) compared to the equal light/dark group. These lost ASVs are likely low in abundance as this effect was not observed in the Shannon and Inverse Simpson metrics (time points 3 & 4 12L:12D vs time points 3 & 4 16L:8D, two‐tailed *t* test, *P* > .05).

### 3.5 Behaviour (OFA, EZM, fecal boli)

We assessed locomotion effects using the Open Field Apparatus (OFA, Figure S9). This test was performed at three different time points: (a) following the acclimation period (baseline), (b) following diet switch (diet effect), and (c) following photoperiod alteration (photoperiod effect). Total distance travelled, immobile time, and number of fecal boli were independently analysed for each period. No significant differences were observed in any of these independent variables during the first period (Figure S9A,D,G).

On the second OFA (Figure S9B,E,H), a 2‐way ANOVA revealed an effect of diet on the number of fecal boli, with lower numbers in the NIF group when compared to the RD group (*F*_1,44_ = 5.80, *P* < .05). No statistically significant effect of photoperiod or interaction was detected on the number of fecal boli during this period. The NIF group also showed higher locomotion (*F*_1,44_ = 13.56, *P* < .001) and less immobile time (*F*_1,44_ = 7.20, *P* < .001) compared to the RD group, as revealed by a 2‐way ANOVA. No significant interaction or photoperiod effect was detected. While photoperiod alone showed no effect, there was a significant interaction between diet and photoperiod (*F*_1,44_ = 4.13, *P* < .05). Tukey's post hoc analysis revealed a significant difference in the 12:12 RD vs 12:12 NIF groups immobile time (*P* < .05).

The same pattern was observed on the third OFA (Figure S9C,F,I). The number of fecal boli was significantly lower in the NIF group compared to the RD (*F*_1,42_ = 6.43, *P* < .05). There was also a significant interaction between diet and photoperiod (*F*_1,42_ = 4.73, *P* < .05). No statistically significant effect of photoperiod alone was detected on fecal boil. Tukey's post hoc analysis revealed a significant difference in the 12:12 RD vs 12:12 NIF groups immobile time (*P* < .05). No significant interaction or photoperiod effect was detected for immobile time during this period. The NIF group also showed higher locomotion (*F*_1,42_ = 8.73, *P* < .001) and less immobile time (*F*_1,44_ = 4.96, *P* < .05), as revealed by a 2‐way ANOVA. No significant interaction or photoperiod effect was detected for locomotion. There was a significant interaction between diet and photoperiod (*F*_1,42_ = 7.11, *P* < .01) for locomotion as well. Tukey's post hoc analysis revealed a significant locomotion difference in the 12:12 RD vs 12:12 NIF groups immobile time (*P* < .01).

### 3.6 Photoperiod‐NIF interaction on endocrine profile (prolactin, GH, and TSH)

A multiplex ELISA analysis was performed to test the effect of shorter active photoperiod and isoflavones on the endocrine profile. Thyroid‐stimulating hormone levels were lower in the NIF diet when compared to the RD (*F*_1,18_ = 12.11, *P* < .005) as revealed by a 2‐way ANOVA. Serum TSH mean levels ± standard error of the mean: RD, 12L12D = 1066.82 ± 87.57; RD, 16L:8D = 1048.42 ± 54.27, NIF, 12L:12D = 862.83 ± 48.98, NIF, 16L8D = 792.48 ± 71.04. No interaction or effect of photoperiod was detected on the TSH levels between groups (*P* > .05). GH and prolactin were also assayed, but no statistical differences (*P* > .05) among groups were found (data not shown).

### 3.7 Liver Nfr2 and PPar‐gamma transcripts

In view of the striking difference in adipocyte size and body weight, we then interrogated the effect of isoflavone and photoperiod duration on liver metabolism. We focused on the nuclear factor erythroid 2 p45‐related factor 2 (Nrf2) due to its involvement in adipogenesis, adipocyte differentiation, and metabolic syndrome. Consistent with the finding of increased adipocyte area and body weight in the NIF diet group, Nrf2 liver transcript levels were significantly elevated in the NIF diet group (*F*_1,20_ = 7.26, *P* < .005), as revealed by a 2‐way ANOVA (Figure S10A). No interaction or photoperiod effects were detected. Similarly, due to the involvement of PPAR‐gamma in obesity and metabolism, we investigated whether the observed increase in adipocyte area and increase in body weight were, in part, reflected by PPAR‐gamma transcript levels (Figure S10B). While we did not detect statistical differences driven by the lack of isoflavones, the longer inactive photoperiod resulted in increased PPAR‐gamma transcript levels compared to the regular photoperiod (*F*_1,20_ = 7.39, *P* < .05). No interaction effect was observed.

## 4 DISCUSSION

Gut microbiome composition is critical for nutrient and food metabolism. Thus, alteration of its makeup may influence the host's metabolism, and ultimately, differentially regulate its energy balance. Our results show that the gut microbiota composition is strongly altered by isoflavone consumption and the length of the photoperiod. These alterations were accompanied by robust metabolic changes suggesting that isoflavones' anti‐lipogenic effects may be driven by the alteration in the composition of the gut microbiota.^28^ A diet free of isoflavones induced significant modifications in the bacterial phyla in vivo, as well as increased indicators of metabolic imbalance.

Previous work by our laboratory and others indicates that isoflavones have a substantial effect on body weight.^18^, ^29^, ^30^ These studies show that animals maintained on an isoflavone‐rich diet are lighter than those on an isoflavone‐free (NIF) diet. This effect seems to be more prominent when the isoflavone concentration is higher.^29^ In the present study, we were able to replicate the bodyweight results, as the mice in the NIF diet showed increased weight gain when compared to animals on a regular diet (RD). Our previous work showed that the RD contains considerable amounts of isoflavones (see Russell et al for exact amounts^18^). In contrast, isoflavones were undetectable in the NIF diet.

Isoflavones are commonly found in soy products, and they are known to mimic estrogen actions.^18^, ^31^, ^32^ They are plant derived nonsteroidal chemicals, which can bind both ERα and ERβ because of their conformational similarity to estradiol.^31^, ^32^ Isoflavones are also found in standard rodent chow.^18^ Studies across different species have shown that dietary isoflavones play a beneficial role in reducing obesity and diabetes markers.^3^, ^4^ Estrogens have a dichotomous effect on body weight and adipose deposition. While estrogens are known to decrease body and adipose tissue weight, they have been shown to increase adipocyte deposition in the aging population,^33^ as well as in pubertal and pregnant women.^34^ However, several studies have shown that the lack of estrogen receptors, or the depletion of estrogen itself, increases adiposity and body weight.^35^, ^36^, ^37^

In the current study, the lack of the estrogenic compound isoflavones, supports the idea that both estrogens and isoflavones are modulators of body weight; however, the mechanism by which this occurs is unknown. Nevertheless, several studies have confirmed the decrease in lipogenesis and weight, and increase in lipolysis caused by isoflavones.^29^, ^38^ This effect may involve the alteration of glucose metabolism by conformational alteration of the membrane‐associated GLUT4 transporter.^39^ Interestingly, we observed an increase in *Glut‐4* levels in the white adipose tissue mRNA transcript levels driven by the NIF diet, perhaps indicating a potential compensational increase of this gene to overcome an insufficient signalling mechanism. We also detected a marked increase in adipocyte size in the animals on the NIF diet compared to the RD group. This finding is consistent with previous studies indicating that treatment with isoflavones decreases adipocyte size.^30^, ^40^ Cederroth et al^40^ present two questions implicating the potential role of ERα and ERβ in mediating the observed metabolic effect of isoflavones: Do isoflavones act (a) directly on the adipocytes or (b) indirectly via the CNS? We argue that a third mechanism may involve changes in the gut microbiome, which ultimately may regulate both central and peripheral sites.^28^

Isoflavones have been shown to act through the peroxisome proliferator‐activated receptor (*Pparg*) to regulate lipids metabolism and insulin sensitization. Genistein and daidzein, in particular, were shown to activate *Pparg* gene expression in in vitro studies,^41^ highlighting genistein as a ligand of *Pparg*.^42^ In the current study, we detected a decrease of *Pparg* in the white adipose tissue in the NIF groups when compared to the regular diet groups, consistent with the increase in body weight and adipocyte area. These data suggest that the increase in body weight and adipocyte area may be due to a compromised lipid metabolism via *Pparg*. Several studies supporting these findings have shown that activation of PPars can improve lipid metabolism and insulin sensitization.^41^, ^43^ Interestingly, the levels of *Pparg* mRNA transcripts were higher in the animals exposed to the shorter active photoperiod, when compared the control 12L:12D, suggesting adipocyte dysfunction similar to recent studies in a rat model of obesity.^44^

The lack of isoflavones also dysregulated the levels of lipoprotein lipase (*Lpl*) in white adipose tissue. Dysregulation of this lipase has been shown to result in obesity and insulin resistance.^45^ We detected an increase in the *Lpl* RNA transcript levels in the animals exposed to the NIF diet when compared to animals in the regular diet. However, the animals in the regular diet that were in the longer inactive photoperiod had lower levels of *Lpl* transcripts. It is possible that the increase in *Lpl* in the NIF group could be leading the increase in body weight, as overexpression of *Lpl* in mice leads to obesity and insulin resistance.^45^ Another gene linked to obesity and type 2 diabetes is *CD36*. *CD36* is a protein that is upregulated in the subcutaneous tissue of obese men and women. There was a significant statistical interaction between diet and the length of the photoperiod in the current study. However, similar to previous studies in which no effects were observed in visceral adipose tissue, no further effects were observed, suggesting limited dynamic changes in non‐subcutaneous adipose tissue.^46^

Another indication of the effects of isoflavones on metabolism was the change in the expression of *Nrf2* and *Pparg* in the liver. *Nrf2* gene expression is involved in the regulation of adipogenesis and insulin resistance. Its involvement with the modulation of antioxidant signalling makes it a potential target for the prevention of metabolic syndrome and cardiovascular disease.^47^ Additionally, adipocyte differentiation is compromised in the absence of *Nrf2*.^48^ Our data suggest that the increase in liver *Nrf2* transcripts may be involved in the adipocyte size anomaly following the removal of isoflavones. We also detected a slight but significant increase in liver *Pparg* transcripts driven by more extended inactive periods. There is extensive literature supporting the role of *Pparg* in food intake and body and fat mass. For example, a *Pparg* agonist increases body and lipid mass, perhaps centrally, through hypothalamic actions.^49^ Thus, the increase of *Pparg* could help explain the synergistic body weight increase in the NIF and the longer inactive period groups.

The composition of the gut microbiome was significantly altered following the removal of isoflavones from the diet. In recent years, there has been a marked increase in the number of studies interrogating the relationship between the gut microbiome with health and disease. Most studies have found a strong association between the gut microbiota composition and several diseases, including obesity.^28^ In humans, for example, the composition of the gut microbiota is altered in obese and diabetic patients,^50^ as well as in those with eating disorders.^51^

The host‐microbiome symbiotic relationship is critical for the maintenance of homeostasis and promoting heath.^52^ For instance, the gut microbiota is required to ferment isoflavones, commonly found in soy food, to more biologically active compounds, which have a relatively higher affinity for ERβ in humans.^53^ Nevertheless, the diet composition can also alter the host‐microbiome interactions, making the microbial community dependent on the host's dietary consumption.^52^ In the present study, the removal of isoflavones caused a switch in the Bacteroidetes‐Firmicutes phyla abundance ratio and an overall drop in diversity levels. Bacteroidetes were higher than Firmicutes in the RD group; however, a switch to the NIF diet caused a reversal of this relationship; Bacteroidetes abundance decreased while Firmicutes abundance dramatically increased in the NIF group. This phenomenon has been previously observed in various diseases, including type‐2 diabetes.^54^, ^55^ With respect to obesity, previous studies have also found a similar change in the ratio of Firmicutes and Bacteroidetes abundance, as observed in our study.^54^, ^56^ Additionally, the observed change in ratio of these two phyla was accompanied by a drop in gut diversity levels once isoflavones were removed from the diet. This gut diversity change has also been previously linked to an isoflavone‐free diet.^57^ Together, these results support the roles for the Firmicutes to Bacteroidetes abundance ratio and diversity metrics as a potential descriptive biomarkers of obesity.^50^, ^58^

In the laboratory, many of the physiological changes related to variations in the circannual rhythm can be achieved by manipulating the length of the day, making it an ideal model. Yet, very limited studies have assessed the effect of the duration of the day on the gut microbiome. Nevertheless, there is a growing number of reports studying the impact of circannual rhythms on metabolism. To the best of our knowledge, no study has investigated the relationship between isoflavones and circannual rhythm on the gut microbiome. In the current study, we interrogated the changes in the gut microbiome driven by the photoperiod‐diet interaction. Most of the effects induced by a long inactive period (16L:8D) were very mild, mainly decreasing the clustering effects of NIF diet. The minor effect of photoperiod could be due to the slight difference between the photoperiods tested. Most of the studies reporting drastic effects on photoperiod alteration utilize protocols assessing both extremes of the spectrum like 16L:8D vs 8L:16D or 19L:5D vs 5L:19D.^11^, ^59^ We decided to execute a protocol that simulates the circannual effect observed in high latitudes vs ones closer to the equator, where the duration of day and night is closer to even.

NIF diet also caused a reduction in TSH. Several laboratories have been asking whether isoflavones may interfere with thyroid function and absorption. In the current work, absence of isoflavones resulted in a reduction of pituitary thyrotropin, TSH, a key player in the hypothalamic‐pituitary‐thyroid axis.^60^ TSH regulates the biosynthesis and secretion of thyroid hormone, and ultimately T3 and T4, which are essential players in the regulation of metabolism. Low TSH could lead to inefficient downstream axis signalling, resulting in low T4. Hypothalamic TRH mRNA in mice treated with an isoflavone‐rich diet^40^ is also compromised. Other reports indicate that isoflavones may inhibit proper thyroid hormone absorption.^61^ Thus, these data indicate that dysregulation of this axis leads to metabolic disorders.

Despite previous reports indicating that mice on the NIF diet consume less food when compared to RD, animals on the NIF diet actually become heavier. Similarly, animals on the NIF diet showed a subtle but significant increase in locomotion. It is possible that locomotion could be increased due to the higher level of estradiol present in the NIF group when compared to the RD group.^62^ In addition to the increase in locomotion, mice in the NIF group showed a decreased number of fecal boli and immobile time, suggesting a reduced response to anxiogenic environments. We have shown that ovariectomized female rats treated with estradiol and isoflavone‐containing diet displayed higher levels of anxiety‐like behaviours when compared to those placed on a NIF diet.^19^ Based on this and previous studies, there is a possibility that isoflavones and estrogens may be interacting to ultimately regulate behaviours. In the above‐discussed study, Russell et al suggest that isoflavones differentially restrict the effects of estradiol, since the anxiogenic effects of estradiol are only present when isoflavones are on board.

This study is not without limitations. Firstly, animals were co‐housed in groups of three. Each cage may have its own microenvironments with similar microbiota induced by cagemate allocoprophagy.^63^ Indeed, we observed that animals from the same cage were more similar to each other (data not shown) than mice from other cages. Despite this observation, we were still able to distinguish microbiome differences between study groups that corroborated with previous literature.^29^, ^38^, ^39^, ^40^, ^64^ Secondly, training accurate machine learning models requires a large number of training observations. Despite the limited number of samples in our study, we were able to discern predictive ASVs that should serve as fodder for future hypothesis‐driven research. Our random forest model was able to predict that much of the variation between the RD and NIF diet groups was attributed to ASVs within the Lachnospiraceae and Clostridiales family of bacteria. Interestingly, the single most predictive ASV belonged to the Muribaculaceae family. This family was highly abundant in the RD group, and was nearly ablated from the gut in the NIF. Similar findings have also been observed in models of induced obesity and type 2 diabetes using the leptin‐deficient mouse (ob/ob) as well as high‐fat diet.^58^ Therefore, the findings that were revealed via machine learning may be helpful when predicting potential metabolic disorders.

In summary, isoflavones have substantial effects on metabolism. Due to isoflavones' potential benefits, several studies have investigated their ability to tackle obesity and type 2 diabetes, hyperlipidemia, hypercholesterolemia, and cardiovascular diseases. In the current study, we show that diet changes the composition of the gut microbiome, perhaps driving the observed metabolic changes. Of note, it is imperative to consider changes in the gut microbiota composition when working with experimental protocols that involve dietary modifications. Based on our and others' data, behavioural and physiological deviations observed in a given experiment could be indeed driven by alterations in the gut microbial community following the diet modification. Finally, it is a safe practice to be conscientious of the composition of the standard diet used in the facilities where animals are housed, since some of the dietary compounds can change seasonally and consequently could interact with the drugs or manipulations studied, ultimately altering the gut microbiome and expected outcomes.

## AUTHOR CONTRIBUTIONS

MGO, BMB, DSM, RJH and TJW conceived and planned the experiments. MGO, BMB, ALR and MC‐C carried out the experiments. MGO, KNB, RCJ. DSM and TJW contributed to the interpretation of results. KGF and KAB‐L conducted the sequencing. RCJ performed the 16S rRNA microbiome analysis. MGO wrote the manuscript. All authors provided feedback and provided integral help in generating this manuscript.

## Supporting information

Fig S1

Fig S2

Fig S3

Fig S4

Fig S5

Fig S6

Fig S7

Fig S8

Fig S9

Fig S10

Table S1

## Data Availability

The data that support the findings of this study are openly available in figshare at https://doi.org/10.6084/m9.figshare.11908410.^65^
