# Supplementary figures and images for: Gut microbiota and metabolic marker alteration following dietary isoflavone‐photoperiod interaction
Source: Endocrinol Diabetes Metab. 2020 Oct 17;4(1):e00190. doi: 10.1002/edm2.190 (PMC7831223; doi:10.1002/edm2.190)

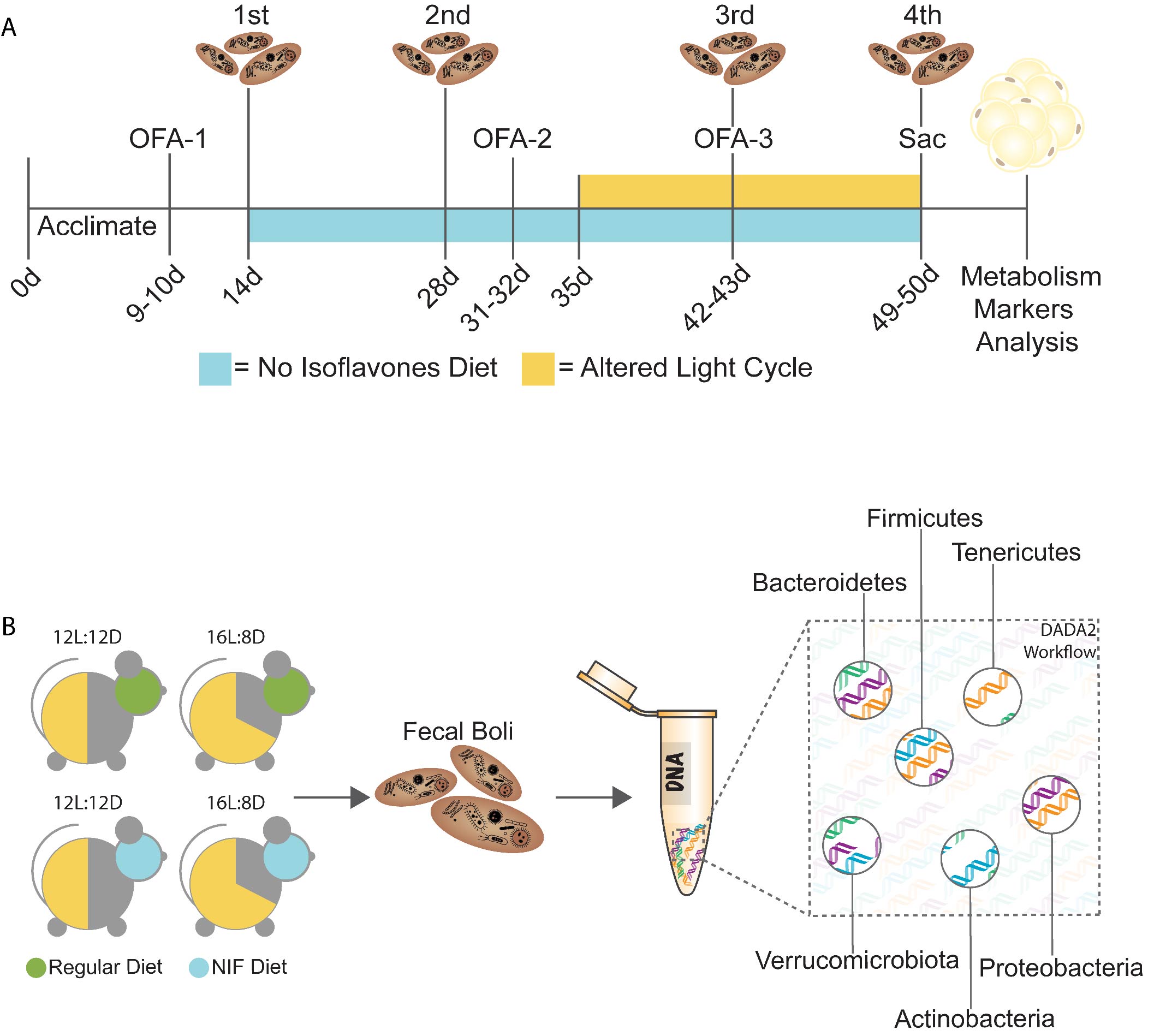

Supplement: Supplementary file 1 — Fig S1 [file EDM2-4-e00190-s001.jpg]

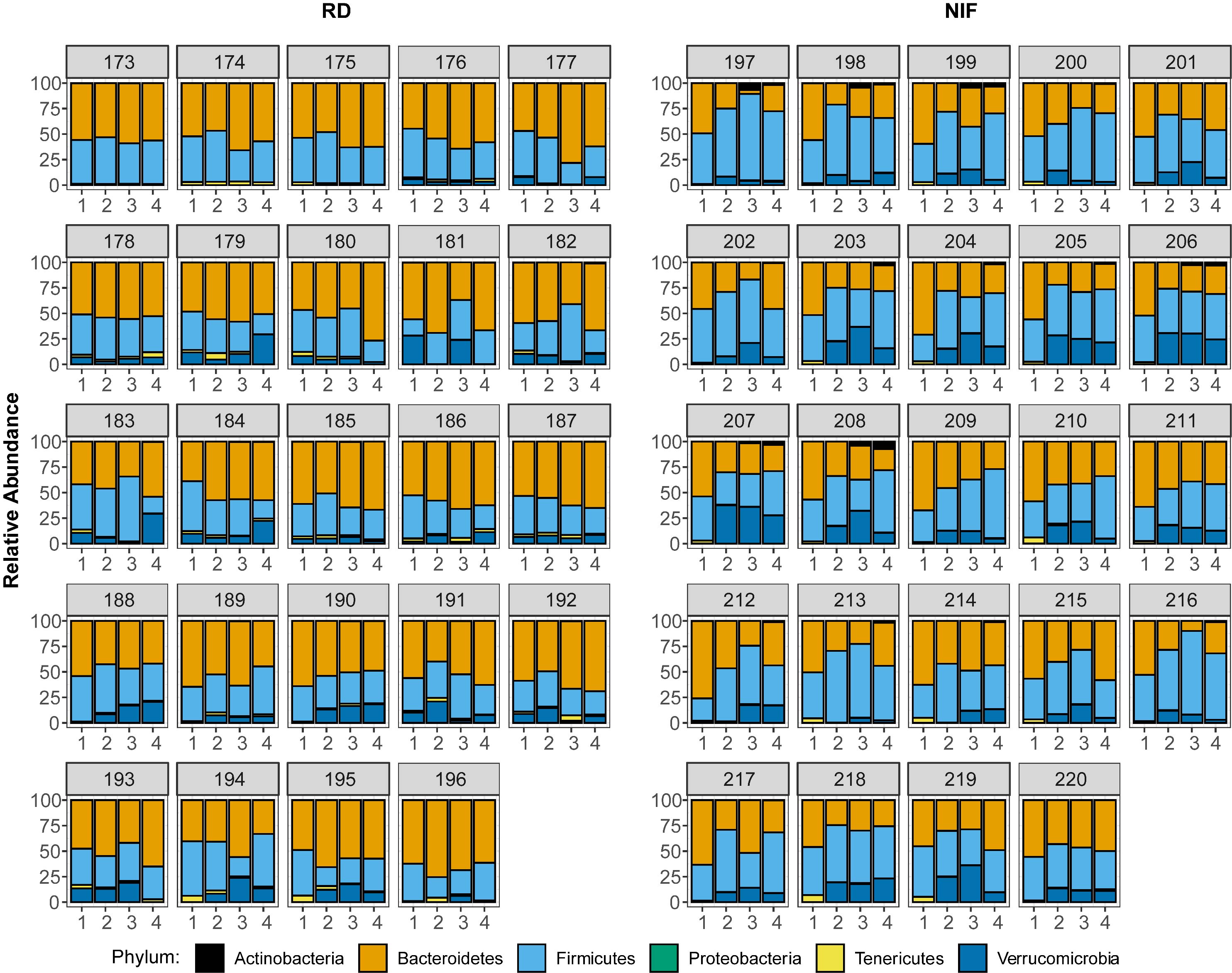

Supplement: Supplementary file 2 — Fig S2 [file EDM2-4-e00190-s002.jpg]

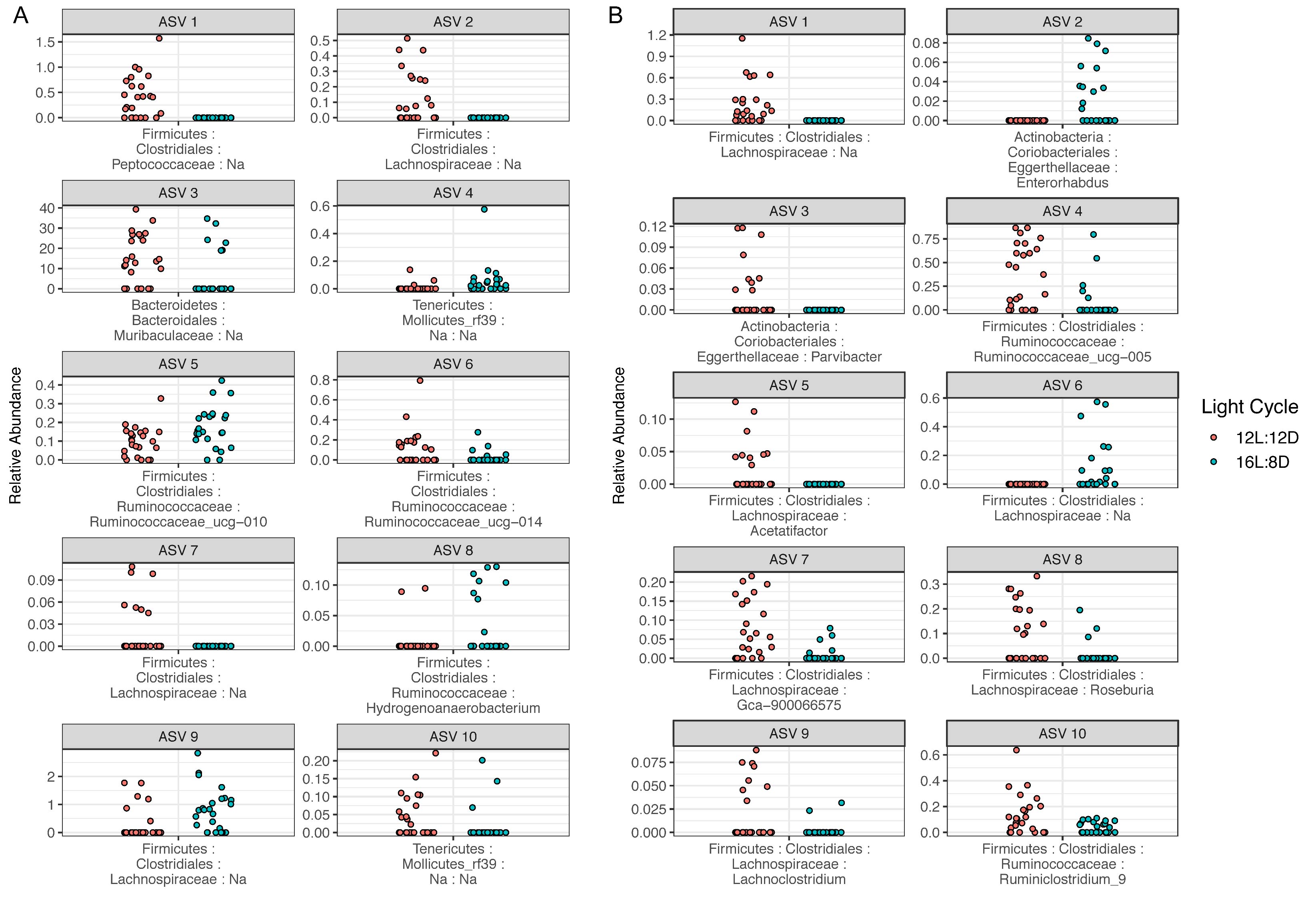

Supplement: Supplementary file 3 — Fig S3 [file EDM2-4-e00190-s003.jpg]

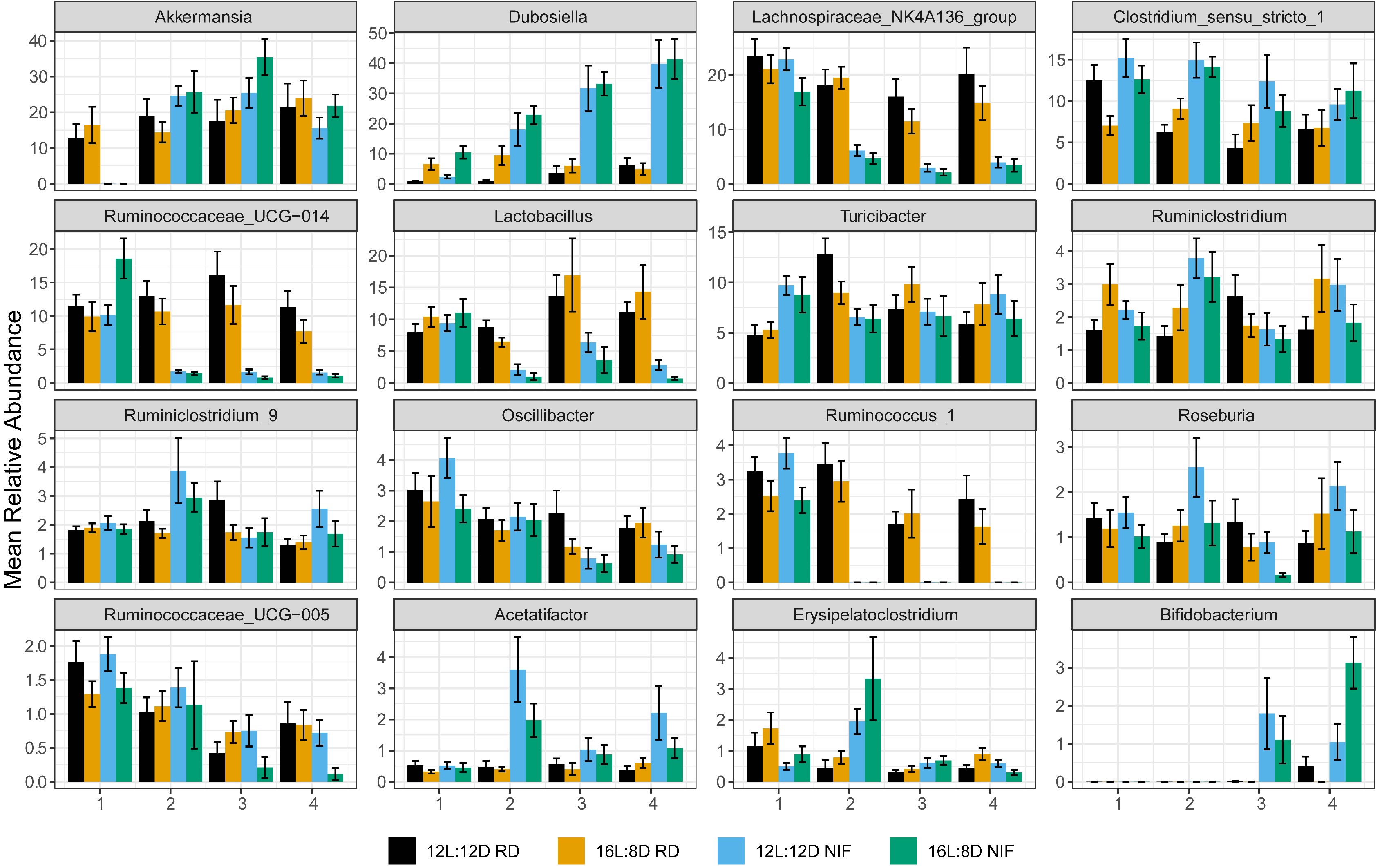

Supplement: Supplementary file 4 — Fig S4 [file EDM2-4-e00190-s004.jpg]

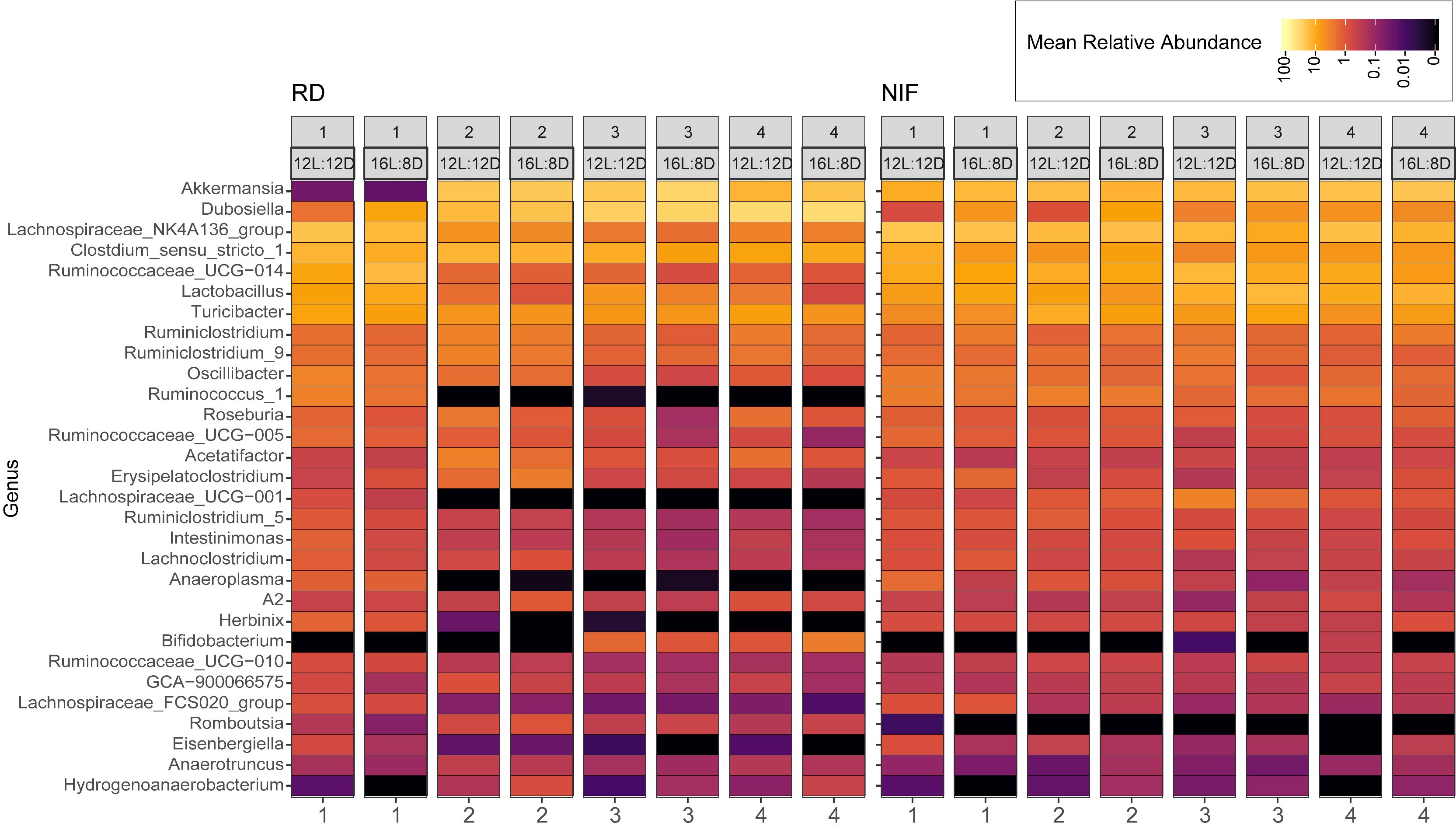

Supplement: Supplementary file 5 — Fig S5 [file EDM2-4-e00190-s005.jpg]

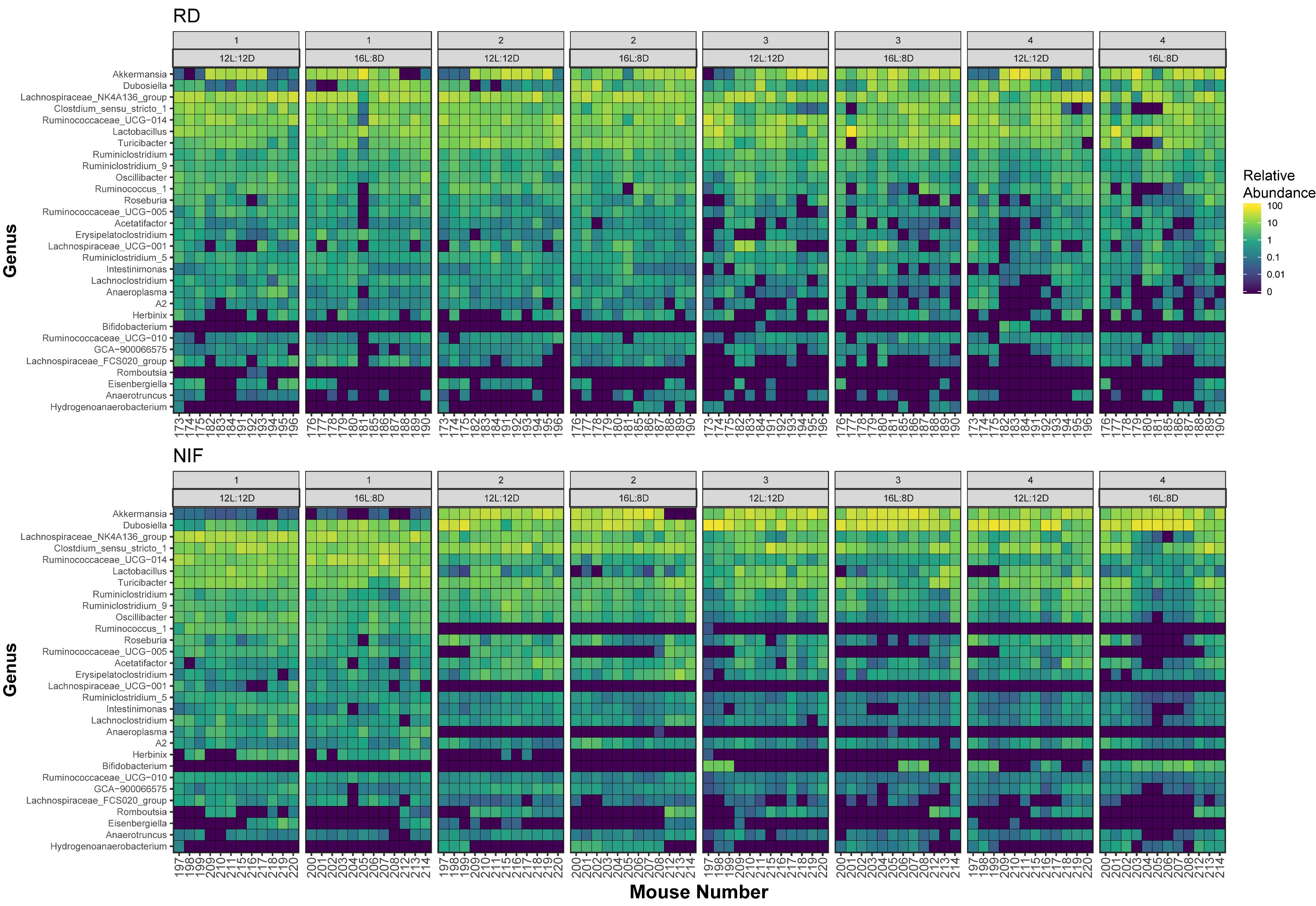

Supplement: Supplementary file 6 — Fig S6 [file EDM2-4-e00190-s006.jpg]

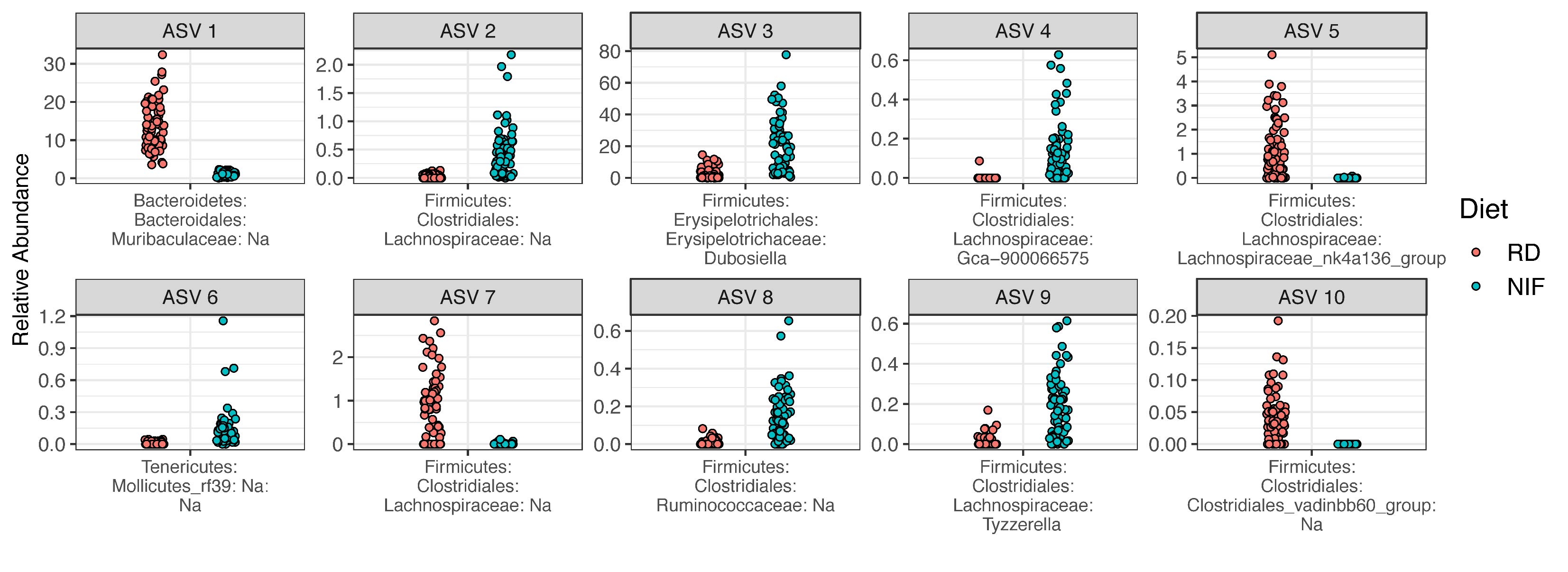

Supplement: Supplementary file 7 — Fig S7 [file EDM2-4-e00190-s007.jpg]

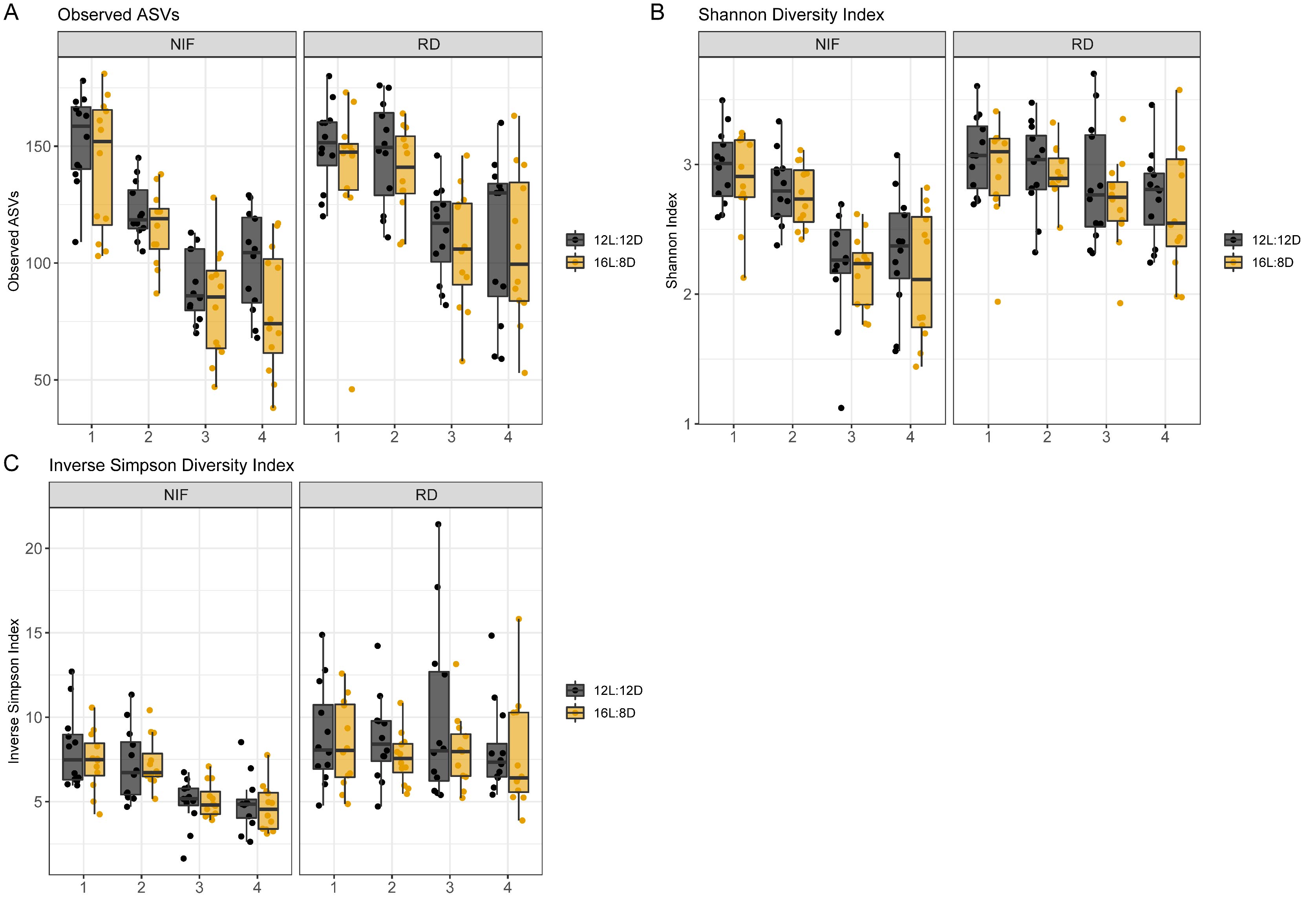

Supplement: Supplementary file 8 — Fig S8 [file EDM2-4-e00190-s008.jpg]

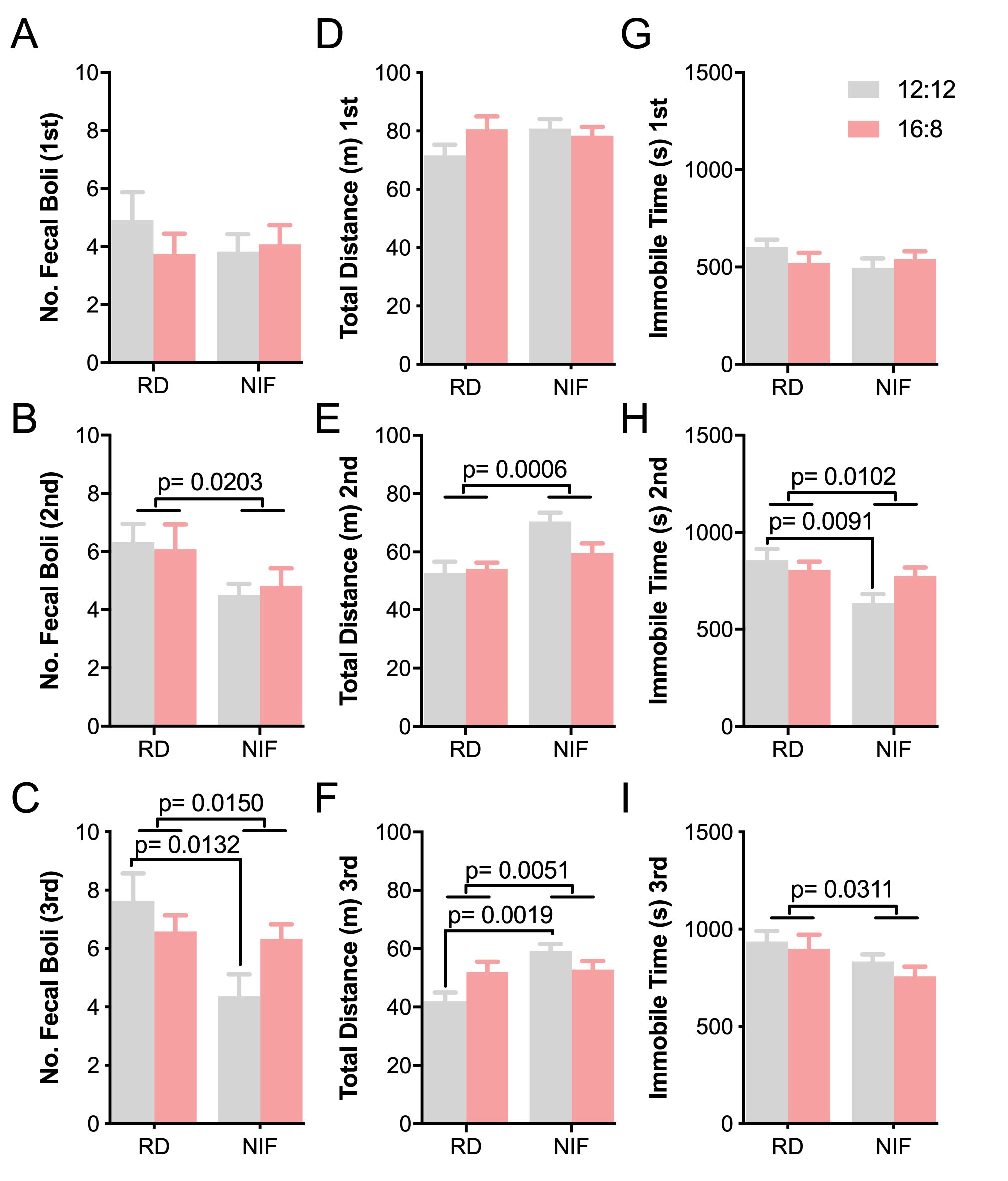

Supplement: Supplementary file 9 — Fig S9 [file EDM2-4-e00190-s009.jpg]

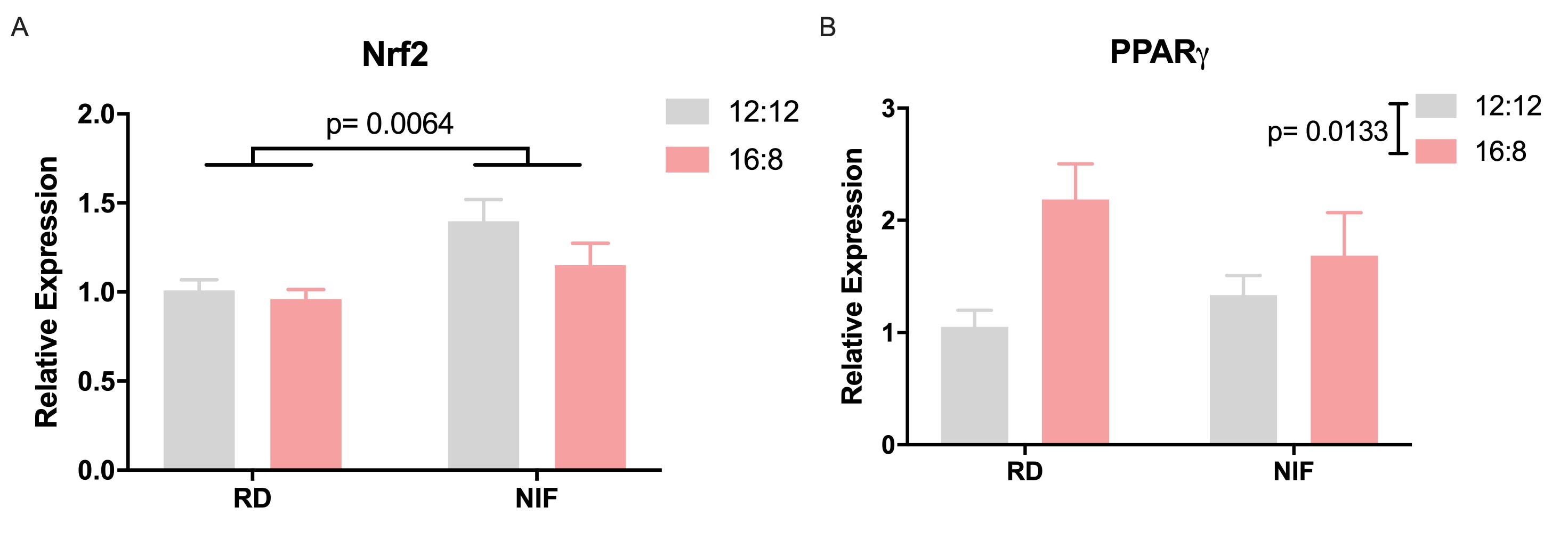

Supplement: Supplementary file 10 — Fig S10 [file EDM2-4-e00190-s010.jpg]
